# Machine learning-based prediction model for lung ischemia-reperfusion injury: insights from disulfidptosis-related genes

**DOI:** 10.3389/fphar.2025.1545111

**Published:** 2025-06-05

**Authors:** Yanpeng Zhang, Jingyang Sun, Yihan Lin, Rongxuan Jiang, Niuniu Dong, Huanhuan Dong, Peng Li, Jinteng Feng, Zijiang Zhu, Guangjian Zhang

**Affiliations:** ^1^ Department of Thoracic Surgery, The First Affiliated Hospital of Xi’an Jiaotong University, Xi’an, Shaanxi, China; ^2^ Key Laboratory of Enhanced Recovery After Surgery of Integrated Chinese and Western Medicine, The First Affiliated Hospital of Xi’an Jiaotong University, Xi’an, Shaanxi, China; ^3^ Biobank, The First Affiliated Hospital of Xi’an Jiaotong University, Xi’an, Shaanxi, China; ^4^ Department of Thoracic Surgery, Gansu Province Central Hospital, Lanzhou, Gansu, China

**Keywords:** IRI, lung transplant, SLC7A11 (xCT), LRPPRC, disulfidptosis

## Abstract

**Objective:**

This study aims to explore potential ischemia-reperfusion injury (IRI) predictive biomarkers related to disulfidptosis following lung transplantation.

**Methods:**

The study utilized datasets from the GEO database, specifically GSE145989 and GSE127003, which include samples of lung cold ischemia and reperfusion following transplantation. Differential expressed analysis and functional enrichment analysis were conducted to identify key genes associated with lung transplant IRI. Multiple machine learning algorithms (Generalized Linear Model, Support Vector Machine, and Random Forest) were applied for joint screening, leading to the construction of a predictive model. The CIBERSORT method was used to assess the infiltration levels of immune cells in lung tissue samples post-transplant. Finally, cell line and animal experiments were carried out to validate the effectiveness and applicability of the model.

**Results:**

A total of 14,592 hub differential-expressed genes were identified, showing significant changes in cold ischemia and reperfusion samples. Using the three machine learning algorithms for joint analysis, a predictive model composed of SLC7A11 and LRPPRC was constructed. This model demonstrated excellent predictive efficacy across multiple datasets, with area under the curve (AUC) values of 0.742 and 0.938, respectively. Additionally, significant differences in neutrophils and macrophages were observed in lung transplant cold ischemia and reperfusion samples. Based on the differential genes associated with disulfidptosis and utilizing the CMap database, we identified two potential drugs targeting IRI: olanzapine and vortioxetine. Ultimately, cell line and animal experiments validated the predictive model’s reliability and potential clinical value, revealing that disulfidptosis presents in IRI, and high SLC7A11 expression promotes IRI, while low LRPPRC expression contributes to its occurrence.

**Conclusion:**

SLC7A11 and LRPPRC can serve as predictive biomarkers for IRI following lung transplantation.

## Highlights

• Innovation: Leveraging three machine learning algorithms (Generalized Linear Model, Support Vector Machine, and Random Forest), we constructed a robust prediction model with high predictive accuracy (AUC = 0.742 in the training cohort and 0.938 in the validation cohort).
• Biological Insight: Experimental findings reveal SLC7A11 and LRPPRC as central regulators in IRI, offering new mechanistic insights into ferroptosis and immune cell dynamics.
• Clinical Translation: Identification of candidate drugs such as olanzapine and vortioxetine via the Connectivity Map database underscores the translational potential of our findings in guiding therapeutic strategies.

## Introduction

For end-stage lung disease, lung transplantation is the only definitive treatment option. Ischemia-reperfusion injury (IRI) is often regarded as a major factor contributing to primary graft dysfunction (PGD), significantly impairing the quality of lung transplant procedures (Chen-Yoshikawa, 2021). The lung is a unique organ characterized by a dual blood supply system composed of pulmonary vessels and the bronchial system. During transplantation, the interruption of bronchial blood supply makes the distal airways more susceptible to ischemic injury. Moreover, during the ischemic phase, the production of reactive oxygen species (ROS) due to various factors leads to severe inflammatory changes, ultimately resulting in cell death in the donor allograft (de Perrot et al., 2003; Capuzzimati et al., 2022). Transcriptomics provides a snapshot of all RNA transcripts present in a cell, organ, or other biological systems (Lowe et al., 2017). By incorporating transcriptomic data, key biomarkers associated with IRI can be identified to form predictive models. This undoubtedly offers transplant surgeons a convenient tool for prediction.

Cell death is one of the primary mechanisms underlying ischemia-reperfusion injury in lung transplantation (Wong and Liu, 2021). Studies have shown that cell death occurs widely during IRI. Wong’s research compared the gene expression profiles of human lung tissues collected at the end of the cold ischemia time (CIT) with those collected from the same donor’s lung after reperfusion, revealing that the enrichment of cell death and inflammation-related gene clusters is one of the most critical events during lung transplantation (Wong et al., 2020). Disulfidptosis, a newly recognized form of cell death (Liu et al., 2023), has yet to be extensively studied in the context of lung transplant IRI. The occurrence of disulfidptosis is dependent on significant depletion of reduced nicotinamide adenine dinucleotide phosphate (NADPH) in environments lacking glucose, leading to the abnormal accumulation of disulfides (Machesky, 2023). The transplantation process itself often involves insufficient glucose supply, compounded by the extensive production of ROS during ischemia-reperfusion, which results in NADPH depletion (Gielis et al., 2015). Consequently, disulfidptosis may also play a role in lung IRI.

A predictive model for IRI following clinical lung transplantation was developed using disulfidptosis-related genes and various machine learning algorithms. Differentially expressed genes (DEGs) in human allograft lung samples before and after transplantation were analyzed using data from the Gene Expression Omnibus (GEO) database. Three machine learning (ML) algorithms were employed to identify biomarkers associated with disulfide cell death from the DEGs, which were then experimentally validated. This study was conducted in accordance with the Declaration of Helsinki.

## Materials and methods

### Data source and acquisition

**FIGURE 1:**
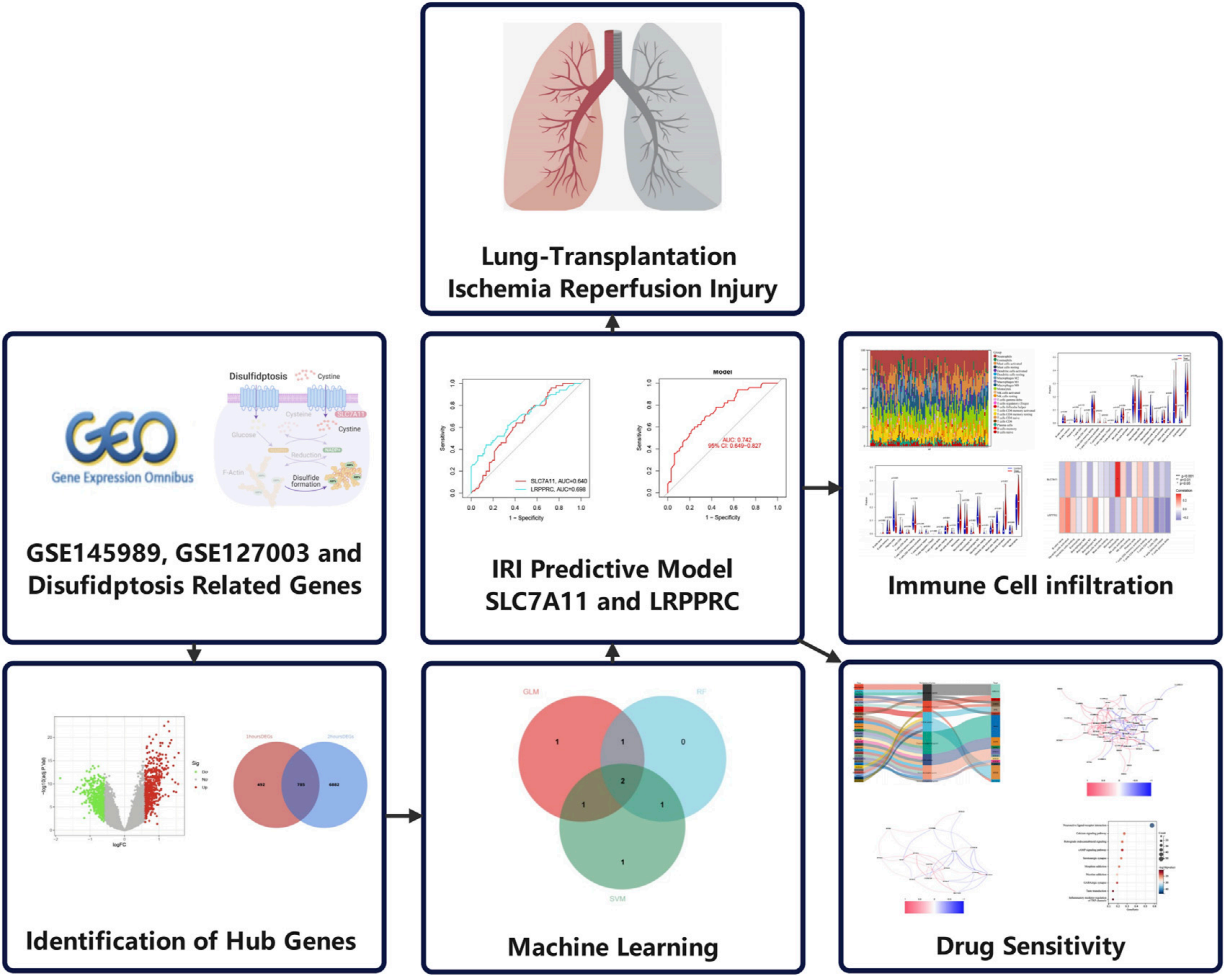
Flowchart of the study.

The overall design of this research suggested in Figure 1.

The datasets included in this study were downloaded from the GEO database (https://www.ncbi.nlm.nih.gov/geo/), specifically GSE145989 and GSE127003. All expression levels were subjected to log2 (x+1) transformation and normalization. Disulfidptosis-related genes acquired from Liu’s research (Liu et al., 2023). The code used for machine learning analysis and model construction is available at [https://github.com/xsddg/mach-learn/blob/bf0b74d1acd4e1962c29741b2bb017b9c06b2b40/geoCRG22.model(1).R#L4].

### Identification of DEDRGs

We utilized the “limma” package and “Wilcoxon test” to compare 1-h and 2-h reperfusion samples in the lung transplantation (LTx) cohort against cold ischemia samples, identifying differentially expressed disulfidptosis-related genes (DEDRGs) through their intersection with known disulfidptosis genes. This analysis revealed genes that are differentially expressed during ischemia and across different reperfusion time points.

### Enrichment analysis

To explore the function of these biomarkers in post-LTx samples, the “c2.cp.kegg.v2022.1. Hs.symbols.gmt” were used as predefined sets to detect significantly enriched pathways with p < 0.05, FDR <25%, and |NES|>1. The top eight gene sets were visualized using “enrichplot” (ver. 1.18.3) in the R package.

### Machine learning

In this study, we utilized three machine learning algorithms—random forest (RF), support vector machine (SVM), and generalized linear models (GLM) to analyze differentially expressed hub genes related to ischemia-reperfusion injury in lung transplantation. For multi-DEDRG selection, simultaneous feature selection was performed using all three methods, with the intersections considered as significant features. Predictive classification models were created based on the selected features using binary classification. Resilient network linear regression was employed to identify relevant differentially expressed genes via the “glmnet” package in R, with a specified regularization parameter λ and a probability threshold of >0. The SVM utilized the ‘e1071’ R package for feature selection, applying a polynomial kernel function. The RF method effectively predicted continuous variables, yielding predictions with enhanced efficacy, sensitivity, and precision.

### Model validation and nomogram construction

The external validation dataset used was GSE127003, which includes paired samples and transcriptomic data from lung transplant recipients after cold ischemia and reperfusion, provided by Toronto General Hospital (Wong et al., 2020). This dataset was incorporated to demonstrate the predictive efficacy of the findings from the training cohort in an external context, thereby enhancing the generalizability of the model.

### Drug sensitivity

Differentially expressed hub DEGs were submitted to the Broad Institute’s Connectivity Map (http://www.broadinstitute.org) database for enrichment analysis (Lamb et al., 2006). The DEGs were used to search for small molecule drugs that may be beneficial for the treatment of IRI. Drugs with negative scores were identified as potentially advantageous for IRI treatment.

### Constructing animal model

Male mice weighing 15–20 g were purchased from the Animal Experiment Center of Xi’an Jiaotong University. The mice were housed in a standard environment at 24°C with a 12-h light/dark cycle, and they had access to food and water *ad libitum*. Every effort was made to minimize the number of animals used and to reduce their suffering. This study was approved by the Animal Experiment Ethics Committee of Xi’an Jiaotong University. Animal care and all experimental procedures were conducted in accordance with the guidelines of the institutional ethics committee.

Specifically, mice were anesthetized with an intraperitoneal injection of water and chloral hydrate (1.5 g/kg). Tracheal intubation was performed, and the mice were ventilated with specific parameters: a tidal volume (TV) of 8 mL/kg and a respiratory rate of 80 breaths per minute (Harvard University, Massachusetts, United States). For the I/R model, a left thoracotomy was subsequently performed, and the left pulmonary hilum was occluded for 90 min. After 90 min of occlusion, the sutures were released to restore blood flow and ventilation to the lung during reperfusion. The sham-operated group underwent a sham surgery, where the left pulmonary hilum was similarly encircled with sutures but not occluded, maintaining bilateral ventilation for a total of 210 min (Wang et al., 2024).

### Cell culture study

BEAS-2B cell line was cultured in Dulbecco’s Modified Eagle Medium (DMEM) supplemented with 10% fetal bovine serum (FBS) and 100 U/mL penicillin/streptomycin solution (GIBCO, Gaithersburg, United States) in a humidified incubator at 37°C with 5% CO_2_.

To generate stable cell lines with knockout of SLC7A11 and LRPPRC, BEAS-2B cells were transfected with sgSLC7A11 and sgLRPPRC constructs, 72 h later, single antibiotic-resistant positive cells were sorted and seeded into a 96-well plate. Surviving knockout clones were screened by immunoblotting using the corresponding antibodies.

### IRI models in BEAS-2B celline

According to Dong’s research (Dong et al., 2021). BEAS-2B cells were cultured with deoxygenated glucose-free Hanks’ Balanced Salt Solution (Beyotime Institute of Biotechnology, Jiangsu, China) and incubated in a hypoxic chamber including 95% N2 and 1% O2 at 37°C for 8 h. After that, cells were incubated with normal culture medium at 37°C for 12 h under normoxic conditions, with or without N-acetyl-cysteine (NAC) (2 mM) for indicated time.

### Cell counting kit-8 assay

To measure cell viability, 3,000 indicated cells were seeded in a 96-well plate per well 24 h before treatment. Upon treatment with the appropriate conditional medium where indicated, each well was replaced with fresh medium containing Cell Counting Kit-8 (CCK8) reagent. After incubation for 60 min at 37°C, each well’s absorbance at a wavelength of 540 nm was measured using a microplate reader.

### Western blotting

As previously described, (Zhang et al., 2024), protein extracts were resolved by SDS-PAGE and transferred to a PVDF membrane (Milipore) using standard techniques. The primary antibodies and concentrations used for Western blotting were: SLC7A11 (1:1,000, CST, 12,691) and LRPPRC (1:1,000, Proteintech, 21175-1-AP).

## Result

### Identification of Hub-DEGs

**FIGURE 2:**
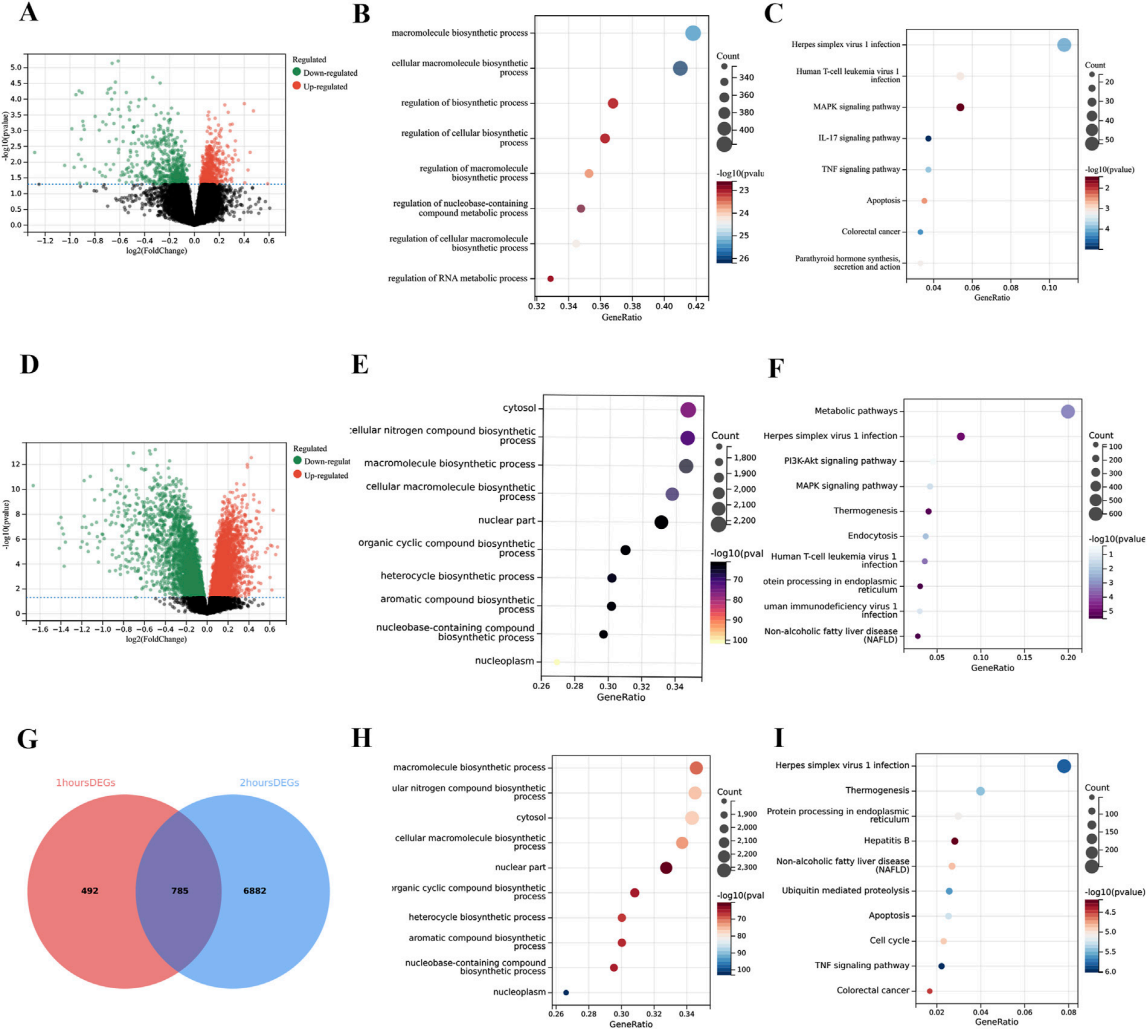
Enrichment analysis of differentially expressed genes (DEGs). **(A)** Volcano plot showing 1,277 DEGs comparing 1-h reperfusion samples with CIT samples. **(B)** Gene Ontology (GO) analysis of DEGs at 1 h. **(C)** Kyoto Encyclopedia of Genes and Genomes (KEGG) analysis of DEGs at 1 h. **(D)** Volcano plot showing 7,667 DEGs comparing 2-h reperfusion samples with CIT samples. **(E)** GO analysis of DEGs at 2 h **(F)** KEGG analysis of DEGs at 2 h. **(G)** Venn diagram illustrating the overlap of DEGs from 1-h and 2-h analyses, identifying hub DEGs associated with ischemia-reperfusion injury (IRI). **(H)** GO analysis of hub DEGs related to IRI. **(I)** KEGG analysis of hub DEGs related to IRI. The X-axis of the volcano plots represents log2 fold change (FC), while the Y-axis represents the log-transformed adjusted P values (p < 0.05).

In the comparison of 1-h reperfusion versus cold ischemia, we identified 1,277 differentially expressed genes. In the comparison of 2-h reperfusion versus cold ischemia, we identified 7,667 differentially expressed genes. By merging the differentially expressed genes from these two time points, we ultimately obtained a total of 8,159 differentially expressed hub genes (Figures 2A,D,G). Regardless of the time point, the DEGs are predominantly enriched in the TNF signaling pathway, apoptosis-related pathways, and cell cycle regulation pathways. Additionally, GO analysis revealed significant enrichment of differentially expressed genes in the context of macromolecule synthesis. Moreover, we observed fluctuations in the number of enriched genes within the TNF signaling pathway, apoptosis-related pathways, and cell cycle regulation pathways (Figures 2B,C). Specifically, there were 77 genes enriched in the TNF pathway at 1 h, while 92 genes at 2 h; this change may reflect the dynamic response of cells to inflammatory signals following CIT treatment. Similarly, in the apoptosis-related pathways, the number of enriched genes was 61 at 1 h, decreasing to 85 at 2 h, suggesting that the regulation of apoptotic signaling may be time-dependent. Similar fluctuations were also noted in the cell cycle regulation pathway, further emphasizing the complex influence of CIT treatment on cellular physiological states. Detailed information on gene enrichment is provided in Supplementary Table S2.

### Selection of DEGs using machine learning algorithms

**FIGURE 3:**
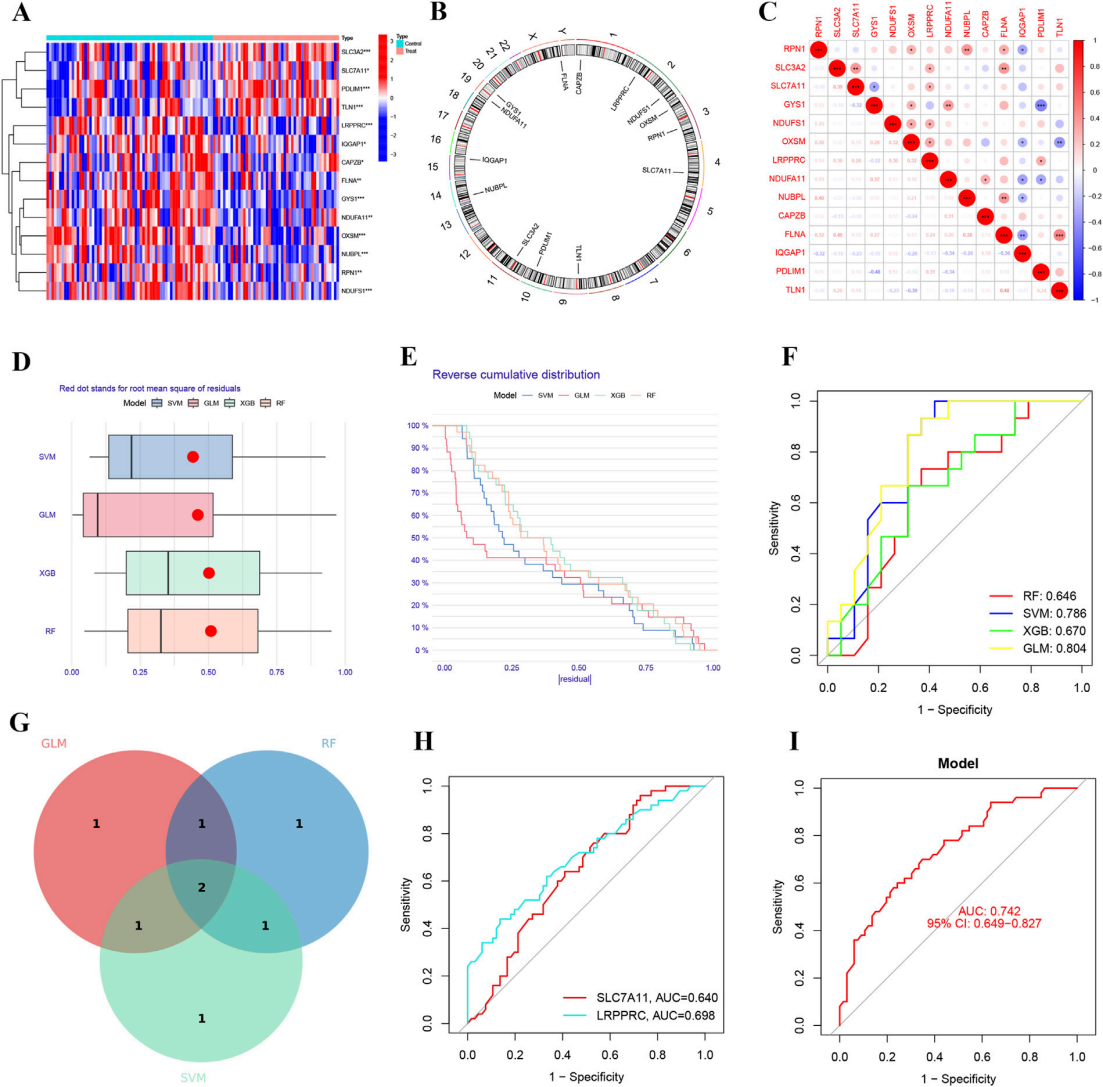
Identification of predictive markers through comprehensive analysis. **(A)** Heatmap of differentially expressed hub genes associated with disulfidptosis. **(B)** Chromosomal locations of the disulfidptosis-related hub genes. **(C)** Correlation analysis among the disulfidptosis-related hub genes. **(D)** Box plots showing sample residuals across four different machine learning models. **(E)** Cumulative residual distribution for each machine learning model. **(F)** Receiver operating characteristic (ROC) analysis of four machine learning models based on 5-fold cross-validation in the testing cohort. **(G)** Venn diagram of genes identified in the IRI model. **(H)** ROC curves demonstrating the predictive efficacy of SLC7A11 and LRPPRC for IRI. **(I)** Predictive performance of the IRI prediction model comprising SLC7A11 and LRPPRC (AUC = 0.742).

In this study, we identified 14 differentially expressed ferroptosis-related genes (Figure 3A). These genes are considered hub ferroptosis genes associated with ischemia-reperfusion injury following lung transplantation. We annotated their positions and created a corresponding chromosomal location map (Figure 3B). There is a broad correlation among these hub ferroptosis genes, with FLNA and TLN1 showing the strongest correlation (cor = 0.48), while SLC3A2 also demonstrated a strong correlation with FLNA (cor = 0.40) (Figure 3C). We employed three machine learning methods to screen these 14 hub genes and evaluated their predictive performance (Figures 3D–F). Ultimately, the results from the GLM, RF, and SVM were presented using a Venn diagram. Upon identifying the overlapping DRGs, we confirmed two biomarkers: LRPPRC and SLC7A11 (Figure 3G). The AUC value for SLC7A11 was 0.640, and for LRPPRC, it was 0.698 (Figure 3H). The ROC curve demonstrated that the machine learning model based on these two biomarkers effectively distinguished between post-LTx and pre-LTx samples (AUC = 0.742) (Figure 3I).

### Nomogram and validation

**FIGURE 4:**
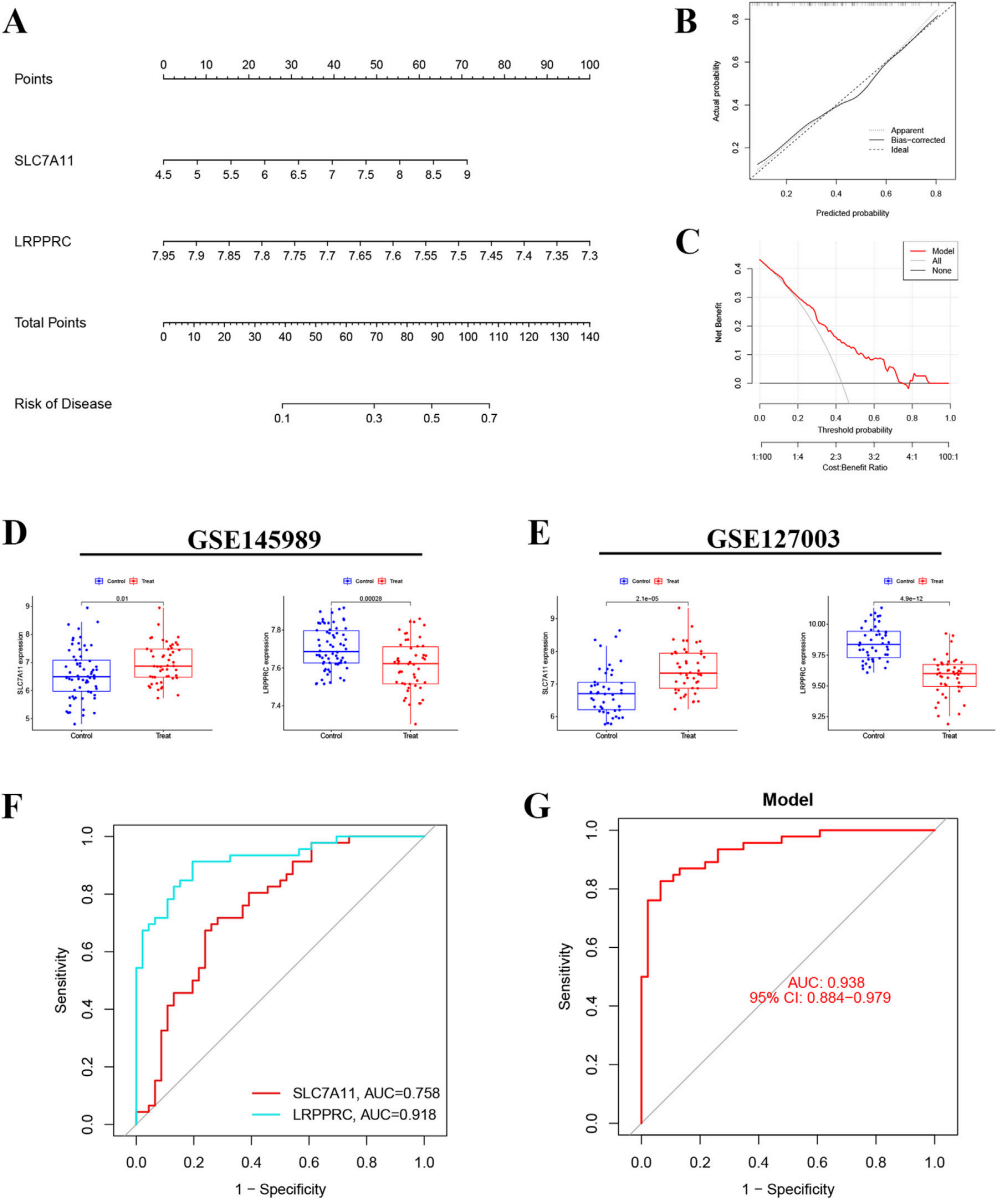
Nomogram construction and model validation. **(A)** Nomogram of diagnostic biomarkers for predicting the occurrence of IRI. **(B)** Calibration curve assessing the predictive power of the nomogram model. **(C)** Decision curve analysis (DCA) curve evaluating the clinical utility of the nomogram model. **(D,E)** Expression levels of SLC7A11 and LRPPRC in datasets GSE145989 and GSE127003. **(F)** ROC curve of SLC7A11 and LRPPRC in GSE145989. **(G)** ROC curve of the IRI prediction model in GSE127003 (AUC = 0.938).

A nomogram was constructed based on the expression of SLC7A11 and LRPPRC, along with calibration and decision curve analyses (Figures 4A–C). This nomogram, integrating the expression levels of SLC7A11 and LRPPRC, allows for specific predictions of IRI-risk in patients. The calibration and decision curve analyses demonstrated that this model possesses strong predictive ability and clinical applicability. Subsequently, we performed a bioinformatics analysis of the expression of genes associated with these two models in the GSE145898 (Figure 4D) and GSE127003 datasets (Figure 4E). The results indicated that SLC7A11 was expressed at higher levels in the reperfusion samples, while LRPPRC had higher expression levels in the non-reperfusion samples. Finally, we validated this model using GSE127003, revealing that the AUC for SLC7A11 was 0.758, while the AUC for LRPPRC was 0.918 (Figure 4F). The overall AUC of our model in GSE127003 was 0.938, which demonstrates the excellent predictive performance of this model (Figure 4G).

### Immune cell infiltration analysis

**FIGURE 5:**
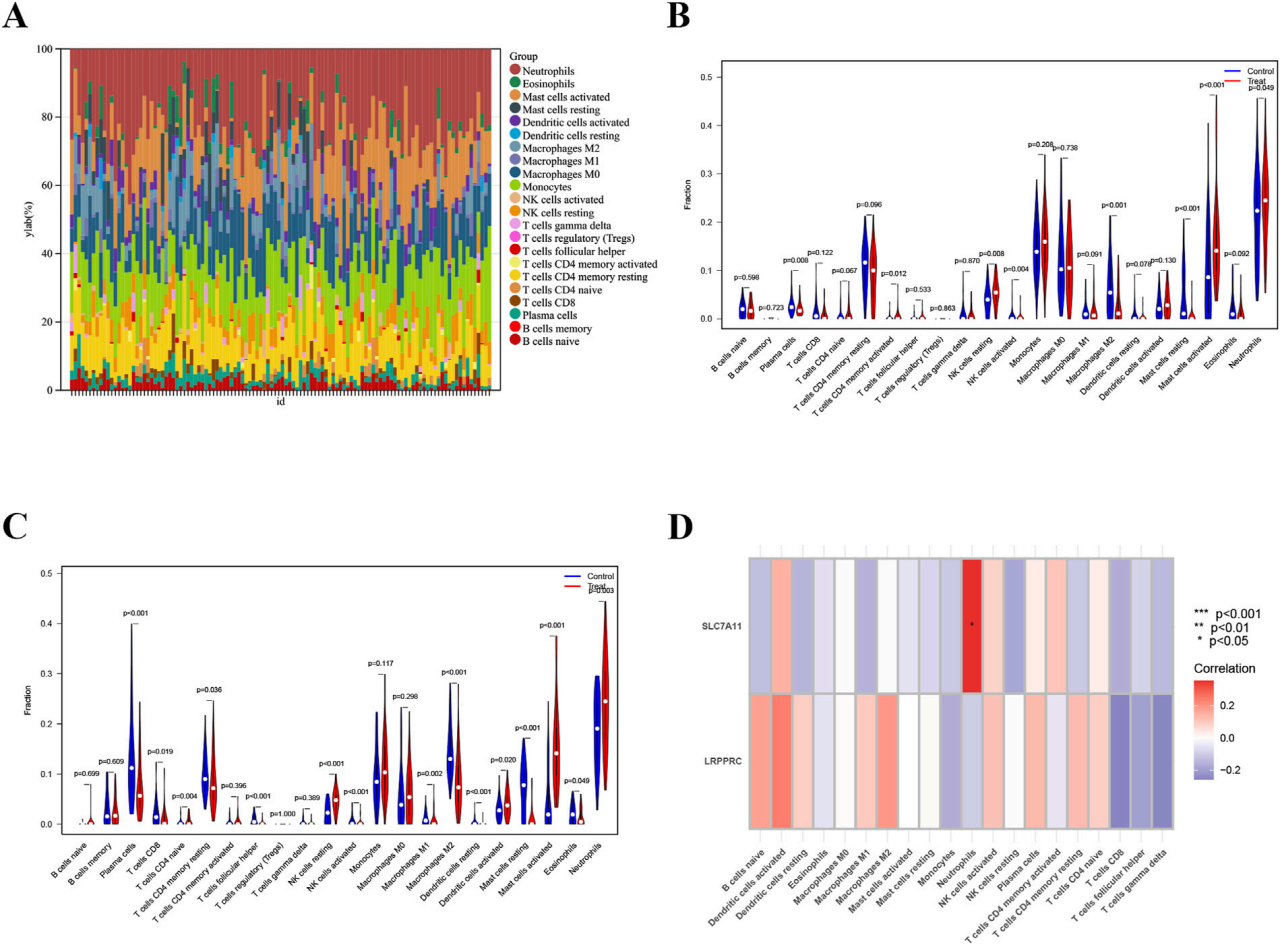
Immune cell infiltration analysis. **(A)** Bar plot depicting the abundance of enriched immune cells in lung transplantation samples. **(B,C)** Violin plots illustrating the distribution of enriched immune cells in training and validation groups. **(D)** Correlation analysis between biomarkers and immune cell populations, with immune cell names on the X-axis and biomarker genes on the Y-axis. Red indicates positive correlations, while blue indicates negative correlations; deeper colors reflect stronger correlations (*p < 0.05, **p < 0.01, ***p < 0.001).

There was an increase and a larger proportion of neutrophil in morphologically diverse samples (Figure 5A). In datasets GSE145989 and GSE127003, a comparison was made between CIT and 2-h reperfusion samples. Significant differences were observed between the two groups in plasma cells, memory CD4^+^ T cells, M2 macrophages, natural killer cells, both activated and resting mast cells, and neutrophils (p < 0.05). In both datasets, the infiltration levels of NK cells, macrophages, and neutrophils showed significant differences between the CIT and reperfusion groups (Figures 5B,C). The expression of SLC7A11 was more closely correlated with neutrophil infiltration, while the expression of LRPPRC showed almost no correlation with the aforementioned immune cells (Figure 5D).

### Drug sensitivity screening

**FIGURE 6:**
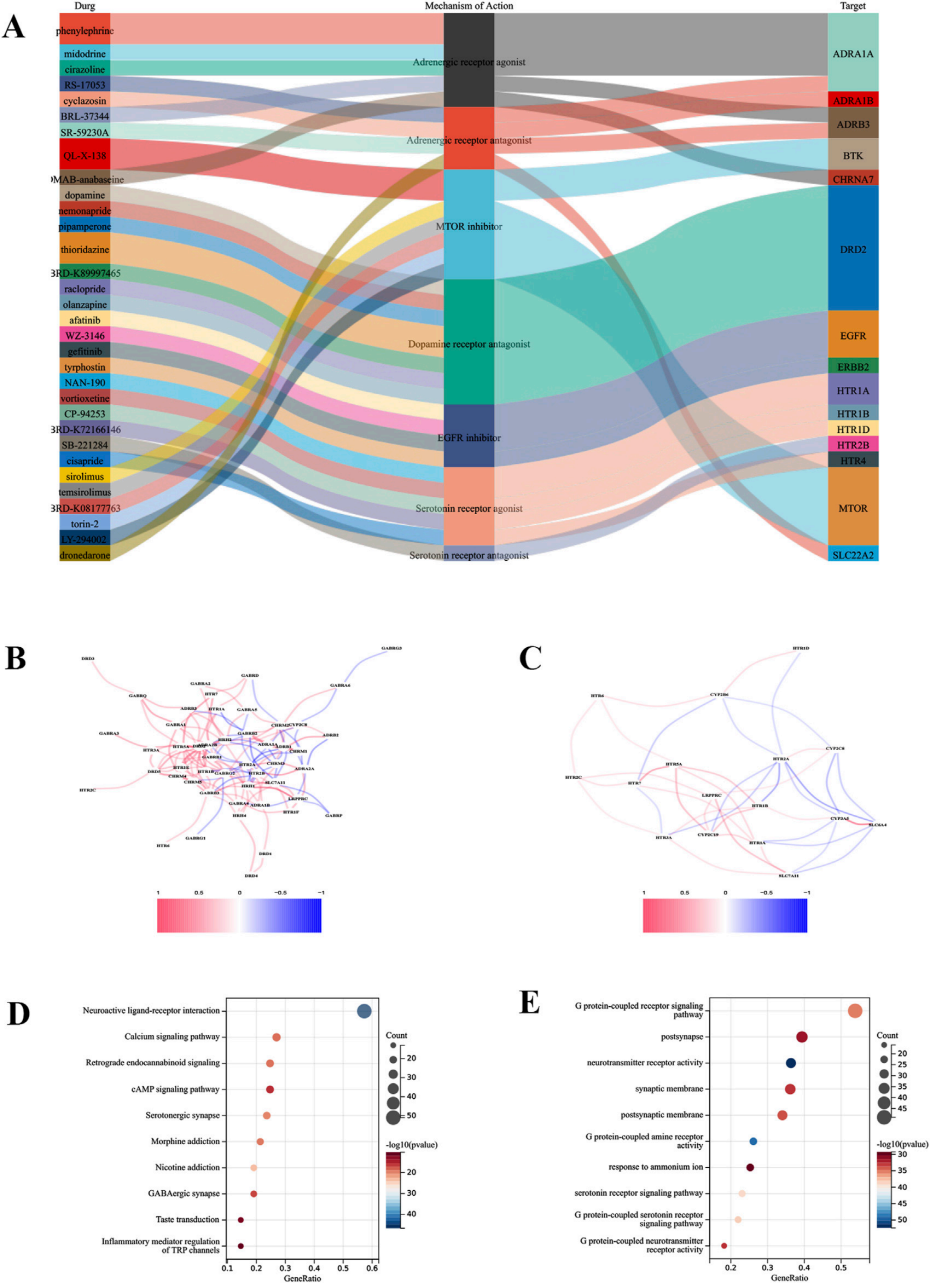
Drug sensitivity analysis. **(A)** Identification of the top five drug classes with the highest sensitivity, based on upregulated DEGs and the CMap database. **(B)** Correlation network between the predictive model and drug targets for olanzapine. **(C)** Correlation network for vortioxetine. **(D)** Enrichment analysis of drug targets using GO analysis. **(E)** Enrichment analysis of drug targets using KEGG analysis.

To identify candidate small molecule drugs for the treatment of IRI, all DEDRGs were classified into upregulated and downregulated groups and uploaded to the CMAP database. Ultimately, five classes of small molecules that may have a modulating effect on IRI following lung transplantation were identified: Adrenergic receptor antagonist, MTOR inhibitor, Dopamine receptor antagonist, EGFR inhibitor, Serotonin receptor agonist (Figure 6A). Further analysis was conducted on the targets of two drugs—vortioxetine and olanzapine—to construct a correlation network with the genes involved in our model (Figures 6B,C). An enrichment analysis of the aforementioned drug targets showed significant enrichment in the Neuroactive ligand-receptor interaction and Calcium signaling pathway. Additionally, GO analysis indicated that the targets of these drugs were significantly enriched in G protein-coupled receptor signaling pathway (Figures 6D,E). This suggests that these drugs may exert their effects on IRI following lung transplantation by participating in the aforementioned biological processes and pathways (Figure 2).

### Experimental validation

**FIGURE 7:**
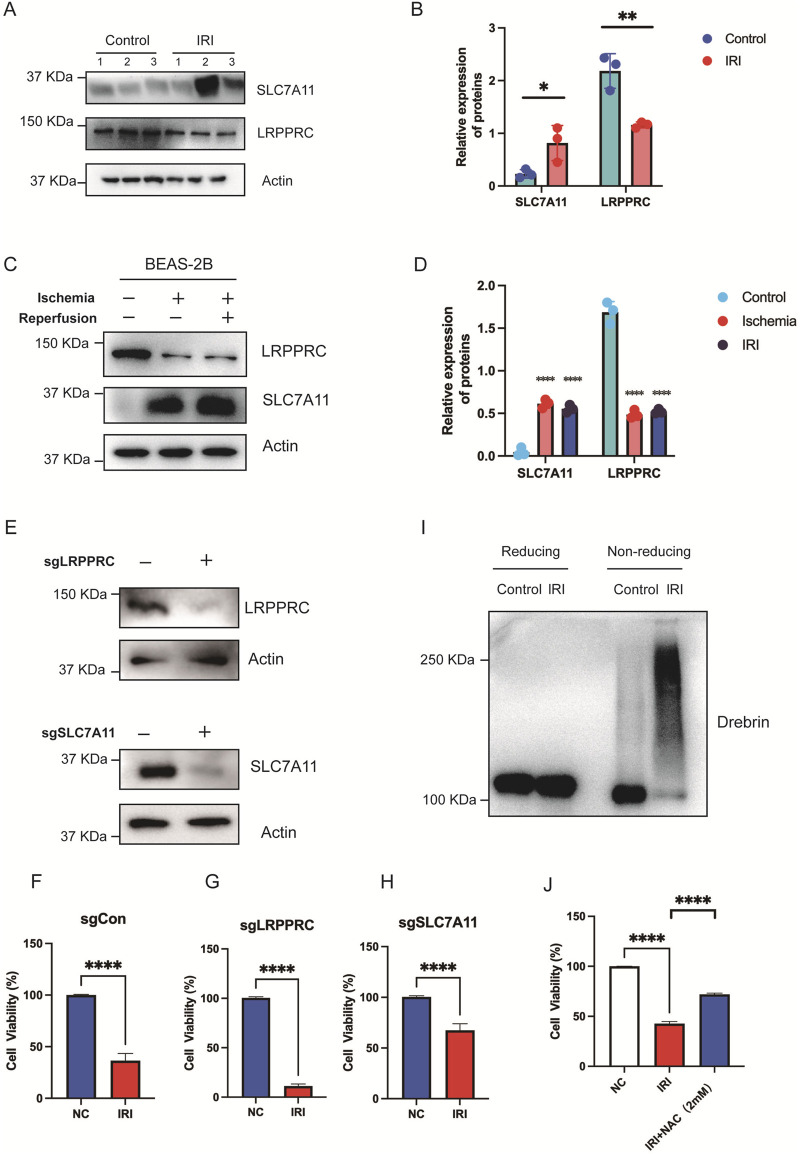
*In vitro* validation of the role of SLC7A11 and LRPPRC. **(A,B)** Immunoblot analysis and quantification of SLC7A11 and LRPPRC expression in lung tissues from normal and IRI mouse models. **(C,D)** Immunoblot analysis and quantification of SLC7A11 and LRPPRC in normal and IRI BEAS-2B cell lines. **(E)** Knockout of LRPPRC and SLC7A11 in stably transfected cell lines was verified. **(F)** CCK-8 assay showing cell viability in normal controls. **(G)** Cell viability in LRPPRC knockout cells. **(H)** Cell viability in SLC7A11 knockout cells. **(I)** The use of reducing and nonreducing immunoblot analysis in lung tissue from mouse models with or without IRI produced the migration retardation of Drebrin. **(J)** Cell viability in NC, IRI, IRI + NAC cells.

In our constructed mouse model of lung IRI, we observed that the expression levels of SLC7A11 in the lung tissue of the IRI group were higher than those in normal lung tissue. However, the expression of LRPPRC was higher in normal tissue compared to IRI tissue, with statistical significance (Figures 7A,B). Further comparisons of the expression differences of these two genes were conducted between the ischemic and reperfusion groups in cell lines. Compared to normal pulmonary epithelial cells, SLC7A11 exhibited elevated expression levels in both the ischemic and reperfusion groups, while LRPPRC only significantly higher in normal group (Figures 7C,D). Subsequently, we established stable knockout cell lines for SLC7A11 and LRPPRC and performed CCK8 assays to assess cell viability (Figure 7E). The results indicated that IRI damage severely compromised cell viability, reducing it to approximately 30% (Figure 7F). After the knockout of LRPPRC, the cell viability in the IRI group was significantly reduced (Figure 7G). However, after knocking out SLC7A11, cell viability significantly increased, restoring to about 70% (Figure 7H). Disulfidptosis is characterized by increased disulfide bonds within cytoskeletal proteins such as Drebrin, which could be monitored by electrophoretic mobility shift under non-reducing conditions (Liu et al., 2023). Indeed, We found that IRI resulted in obvious migration retardation of Drebrin in lung tissue from IRI mouse models (Figure 7I). Furthermore, disulfidptosis inhibitor NAC significantly rescued cell viability caused by IRI (Figure 7J). These findings, in conjunction with the bioinformatics analyses and animal experiment results, suggest that SLC7A11 and LRPPRC may play a promoting role in lung IRI damage. This provides strong experimental support for the predictive stability of our model, indicating that the measurement of SLC7A11 and LRPPRC expression could serve as a predictor of IRI following lung transplantation.

## Discussion

This study integrates disulfidptosis-related genes and machine-learning algorithms to develop personalized and precise predictive model for IRI in patients following lung transplantation. The specific conclusions of the study are as follows: First, a predictive model for post-transplant IRI was constructed based on SLC7A11 and LRPPRC, demonstrating strong predictive performance. Second, SLC7A11 may influence neutrophil infiltration, which in turn affects lung IRI. Additionally, through the screening of disulfidptosis-related genes, two potential therapeutic agents, olanzapine and vortioxetine, were identified, which may have beneficial effects on IRI following lung transplantation. Finally, experimental validation confirmed that disulfidptosis occurs in IRI, SLC7A11 and LRPPRC promote lung IRI, indicating that they could serve as potential therapeutic targets for IRI after lung transplantation.

Reperfusion injury is unavoidable post-transplantation (Chen-Yoshikawa, 2021). Although some methods validated in animal models have shown promise in reducing the severity of IRI, it continues to pose significant challenges for transplant surgeons in clinical settings (Almeida et al., 2020; Gouchoe et al., 2024). Integrating various machine learning approaches can consolidate the key features identified by different methods. By combining multiple algorithms, we can develop a consensus model for IRI predicting, simplifying the model and enhancing its portability by reducing the dimensionality of variables-an advantage of comprehensive machine learning analysis.

The activation of disulfidptosis may require three criteria: (1) high expression of SLC7A11; (2) glucose deprivation conditions that block glucose metabolism and promote the production of reduced NADPH through the pentose phosphate pathway (PPP) (Gouchoe et al., 2024); and (3) abnormal disulfide bond formation between actin cytoskeletal proteins. When all these conditions are met, excessive accumulation of disulfide bonds occurs, leading to the formation of disulfide bonds between actin cytoskeletal proteins, actin contraction, and detachment from the plasma membrane, ultimately resulting in cell shrinkage and death (Liu et al., 2023). A characteristic of IRI is the rapid accumulation of reactive oxygen species (ROS) shortly after reperfusion, accompanied by an increase in the activity of ROS-generating enzymes (Chatterjee et al., 2014). NADPH oxidase is the only known enzyme responsible for producing ROS, and it is widely expressed in alveolar epithelial cells (Panday et al., 2015). During IRI, the production of ROS consumes a significant amount of NADPH, leading to a decline in the cell’s antioxidant capacity and exacerbating oxidative stress. Our experimental data have confirmed that SLC7A11 is highly expressed in reperfusion samples, consistent with sequencing analysis results. Furthermore, the knockout of SLC7A11 significantly restored cell viability after IRI damage, suggesting that SLC7A11 may influence lung IRI. However, whether it impacts IRI through the regulation of disulfidptosis still requires further experimental validation. By using cell experiments, we witnessed the occurrence of disulfidptosis during IRI, and disulfidptosis inhibitor NAC could partially rescue the cell viability. According to Liu’s study, the inactivation of LRPPRC synergistically induces cell death in conjunction with glucose starvation (Liu et al., 2023). Additionally, our study found lower levels of LRPPRC in cell lines and animal models undergoing IRI. This indicates that LRPPRC may be involved in disulfidptosis in the IRI environment by affecting glucose metabolism. In fact, after lung transplantation, the ischemic process creates a naturally glucose-deprived environment, which undoubtedly provides the necessary conditions for the occurrence of disulfidptosis. In our study, both cell lines and animal models of IRI were used to support the occurrence of disulfidptosis under IRI. Therefore, the potential mechanism of disulfidptosis in IRI during lung transplantation should be further studied in the future.

In the study of IRI, the significant increase in DEGs with prolonged reperfusion time provides crucial insights into the molecular mechanisms underlying I/R injury. Notably, the enrichment analysis of the MAPK signaling pathway at both the 1-h and 2-h reperfusion time points highlights its critical role in both early and later stages of injury. Research indicates that erythropoietin (EPO) can alleviate acute lung injury caused by I/R by blocking the p38 MAPK signaling pathway (Jia et al., 2021). Furthermore, inhibiting p38 MAPK can reduce the high permeability of the blood-gas barrier, thereby mitigating lung ischemia-reperfusion injury (Wang et al., 2020). These findings confirm the research value of the MAPK signaling pathway in lung I/R injury, suggesting that attention should be paid to the regulation of this pathway in clinical interventions, allowing for targeted protective measures at different time points to effectively reduce the extent of injury.

During the cold ischemia and reperfusion phases, the immune cell profile exhibits differences, primarily involving natural killer (NK) cells, macrophages, mast cells, and neutrophils. The infiltration of M2 macrophages during the cold ischemia phase is significantly greater than that during the reperfusion phase. Quercetin has been shown to upregulate M2 markers and downregulate M1 markers, with this effect mediated via the PI3K/Akt/NF-κB signaling pathway (Li et al., 2023). Tetrahydrocurcumin (THP) induces the polarization of M1 macrophages to M2 by inhibiting the TLR4/NF-κB/NLRP3 signaling pathway, thereby alleviating acute lung injury caused by limb ischemia-reperfusion in rats (Li et al., 2023). Furthermore, we observed a significant correlation between the expression of SLC7A11 and neutrophil infiltration. IRI-mediated damage is associated with neutrophil infiltration, and inhibiting this infiltration can prevent IRI after transplantation (Shepherd et al., 2022). It may be possible to influence neutrophil infiltration and thereby control IRI damage by suppressing SLC7A11 expression. However, these conclusions are based on a single dataset, and the specific changes in immune cells related to the expression of model-associated genes in lung transplant samples require validation through multiple transcriptomic datasets and experiments. Additionally, the conclusions derived from the CIBERSORT algorithm are estimations based on gene expression, and their precision remains insufficient.

In this study, we identified several potential candidate drugs through the screening of genes associated with disulfidptosis Olanzapine is an antipsychotic medication widely used for the treatment of schizophrenia and bipolar disorder, and its neuroprotective potential has garnered increasing attention. Research has indicated that olanzapine-induced hypothermia provides protective effects against renal injuries in rats subjected to asphyxiation-induced cardiac arrest (Tungalag et al., 2022). Vortioxetine, a novel antidepressant, has also been shown to possess neuroprotective effects. Studies have reported that vortioxetine mitigates neuronal injury caused by ischemia-reperfusion damage by inhibiting the PERK/eIF2α/ATF4/CHOP signaling pathway (Emam et al., 2021). Furthermore, vortioxetine may influence ferroptosis by reducing the overexpression of NADPH oxidase 2 (Caruso et al., 2021), thereby decreasing the production of reactive oxygen species (ROS). In recent years, the research strategy of “repurposing old drugs” has emerged as a prominent area of interest. Our IRI prediction model reveals significant interactions with the targets of these two drugs, suggesting the presence of potential unknown mechanisms and research gaps. In the future, our research team will conduct in-depth investigations into the drugs identified in this study, aiming to provide new theoretical foundations and practical guidance for the treatment of cell death and IRI damage following lung transplantation.

Our study has several limitations. First, the limited sample size included in our validation study may restrict the generalizability of the results. Therefore, a larger sample size is necessary to comprehensively identify key genes associated with lung IRI. Additionally, for machine learning applications, it is essential to train predictive models using multi-center sequencing data to improve their predictive capabilities. A limitation of our study is the absence of detailed clinical background information, including the type of pulmonary disease, patient age, and concurrent medications. This may introduce potential confounding factors and limit the generalizability of the identified biomarkers. Future studies involving well-annotated clinical cohorts are warranted. Finally, while the disufidptosis-related genes identified in our study are based on existing literature, continuous exploration and updates are required to refine and expand this knowledge base.

## Conclusion

In conclusion, we identified two final hub genes as disulfidptosis-related biomarkers for IRI during lung transplantation using various machine learning algorithms. SLC7A11 and LRPPRC were confirmed to be highly expressed after reperfusion in both animal and cell experiments, providing new insights into the role of immune cells in IRI during lung transplantation.

## Data Availability

The raw data supporting the conclusions of this article will be made available by the authors, without undue reservation.
